# How to review and assess a systematic review and meta-analysis article: a methodological study (secondary publication)

**DOI:** 10.3352/jeehp.2023.20.24

**Published:** 2023-08-27

**Authors:** Seung-Kwon Myung

**Affiliations:** 1 Department of Cancer AI & Digital Health, National Cancer Center Graduate School of Cancer Science and Policy, Goyang, Korea; 2 Cancer Epidemiology Branch, Division of Cancer Data Science, National Cancer Center Research Institute, Goyang, Korea; 3 Department of Family Medicine and Center for Cancer Prevention and Detection, National Cancer Center Hospital, Goyang, Korea; Hallym University, Korea

**Keywords:** Evidence-based medicine, Meta-analysis as topic, Patient care, Research design, Systematic review as topic

## Abstract

Systematic reviews and meta-analyses have become central in many research fields, particularly medicine. They offer the highest level of evidence in evidence-based medicine and support the development and revision of clinical practice guidelines, which offer recommendations for clinicians caring for patients with specific diseases and conditions. This review summarizes the concepts of systematic reviews and meta-analyses and provides guidance on reviewing and assessing such papers. A systematic review refers to a review of a research question that uses explicit and systematic methods to identify, select, and critically appraise relevant research. In contrast, a meta-analysis is a quantitative statistical analysis that combines individual results on the same research question to estimate the common or mean effect. Conducting a meta-analysis involves defining a research topic, selecting a study design, searching literature in electronic databases, selecting relevant studies, and conducting the analysis. One can assess the findings of a meta-analysis by interpreting a forest plot and a funnel plot and by examining heterogeneity. When reviewing systematic reviews and meta-analyses, several essential points must be considered, including the originality and significance of the work, the comprehensiveness of the database search, the selection of studies based on inclusion and exclusion criteria, subgroup analyses by various factors, and the interpretation of the results based on the levels of evidence. This review will provide readers with helpful guidance to help them read, understand, and evaluate these articles.

## Graphical abstract

[Fig f5-jeehp-20-24]

## Introduction

### Background

Borenstein et al. [1] begin the preface of their textbook entitled Introduction to Meta-Analysis by giving an example of sudden infant death syndrome (SIDS). Dr. Benjamin Spock, who was considered to be one of the most famous and influential American pediatricians of the 20th century, wrote, “I think it is preferable to accustom a baby to sleeping on his stomach from the beginning if he is willing” in his book entitled *The Common Sense Book of Baby and Child Care*, which is one of the best-selling books of the 20th century. About 50 million copies of this book were sold between the 1950s and 1990s [1]. Many pediatricians also gave similar advice at the time. During the same period, more than 100,000 babies died of SIDS [1]. In the meantime, in a systematic review and meta-analysis of 40 observational studies published in 2005, Gilbert et al. [2] reported that front sleeping, compared with back sleeping, statistically significantly increased the risk of SIDS by about 3 times by 1970 (pooled odds ratio [OR], 2.93; 95% confidence interval [CI], 1.15 to 7.47). The authors concluded that a “systematic review of preventable risk factors for SIDS from 1970 would have led to earlier recognition of the risks of sleeping on the front and might have prevented over 10,000 infant deaths in the United Kingdom and at least 50,000 in Europe, the United States, and Australasia” [2]. This example shows the importance of systematic reviews and meta-analyses in the field of medicine.

Recently, systematic reviews and meta-analyses have emerged as a frequently used and central method in many fields of research such as psychology, pedagogy, criminology, business, ecology, and other scientific fields, as well as medicine.

### Objectives

This review aims to help the readers of this journal learn about what systematic reviews and meta-analyses are and how to review and evaluate those papers, especially in the field of medicine.

## Ethics statement

As a literature-based study, approval by the Institutional Review Board and informed consent were not required.

## What is a systematic review?

At a simple level, research articles can be divided into 2 types: original research articles and review articles. Original research articles are the most common type of research articles published in scientific journals, and they report the research question, methods, results, and conclusions of an original study actually conducted and written by the author(s). These articles are classified as primary literature [3,4]. On the contrary, review articles report a summary and/or synthesis of the research findings from the existing published literature on a certain topic, and they are classified as secondary literature [4]. Review articles can be further divided into 2 types: narrative review articles and systematic review articles. A narrative review, also known as a traditional nonsystematic review, is a subjective overview and broad qualitative summary of the current knowledge on a certain topic by an expert using selected literature without prespecified or documented selection criteria and methods to support their conclusion [5]. On the contrary, according to the glossary of terms in the Cochrane Collaboration updated in 2005 [6], a systematic review is “a review of a clearly formulated question that uses systematic and explicit methods to identify, select, and critically appraise relevant research, and to collect and analyze data from the studies that are included in the review. Statistical methods (meta-analysis) may or may not be used to analyze and summarize the results of the included studies.” The main difference between narrative reviews and systematic reviews is that systematic reviews answer a clearly defined, narrow question through explicit search strategies with predefined selection criteria and data extraction and appraisal in a structured way, with or without a quantitative method such as meta-analysis [7].

## What is a meta-analysis?

The idea of dealing quantitatively with various individual observations emerged in the 17th century, when the French mathematician Blaise Pascal developed mathematical ways of handling games of chance in gambling. Although Karl Pearson’s “Report on Certain Enteric Fever Inoculation Statistics” in 1904 is considered to be the first meta-analysis, it was not until 1976 that the term “meta-analysis” was coined by Gene V. Glass, who is an American statistician and educational psychologist [8,9]. In his article published in the journal Education Researcher in 1976, he used the term “meta-analysis” to refer to “analysis of analyses,” specifically referring to “the statistical analysis of a large collection of analysis results from individual studies for the purpose of integrating the findings” [10]. That is, a meta-analysis is a quantitative statistical analysis combining individual results to estimate the common or mean effect. Since then, meta-analyses have been conducted in various fields of study, such as psychology, sociology, pedagogy, and medicine, and meta-analysis has come to be seen as an important component of a systematic review. In particular, systematic reviews and meta-analyses generally provide the highest level of evidence in evidence-based medicine (EBM), supporting the development and revision of clinical practice guidelines, which are recommendations for clinicians when caring for patients with specific diseases and conditions [11].

## Evidence-based medicine, the levels of evidence pyramid, and systematic reviews with meta-analyses

In 1972, Archie Cochrane, who was a Scottish doctor and is now known as one of the pioneers of modern clinical epidemiology and EBM, articulated the criticism that many practices in medicine that had previously been believed to be effective lacked evidence from randomized controlled trials in his book entitled Effectiveness and Efficiency [12]. Since then, the term “evidence-based” began to be used regarding clinical practice guidelines, and several papers discussing evidence-based guidelines and policies were published in the *Journal of the American Medical Association* between 1990 and 1997. The term “evidence-based medicine” was first used by Gordon Guyatt of McMaster University in 1991 [13]. In their editorial published in *British Medical Journal* in 1996, Sackett et al. [13] clearly defined EBM as “the conscientious, explicit and judicious use of *current best evidence* in making decisions about the care of individual patients. The practice of EBM means integrating individual clinical expertise with the best available external clinical evidence from systematic research” (emphasis added) [14]. Since the publication of this editorial, EBM has been the basis for the development of clinical practice guidelines, which provide doctors recommendations for the medical treatment of various diseases.

**Fig. 1.**
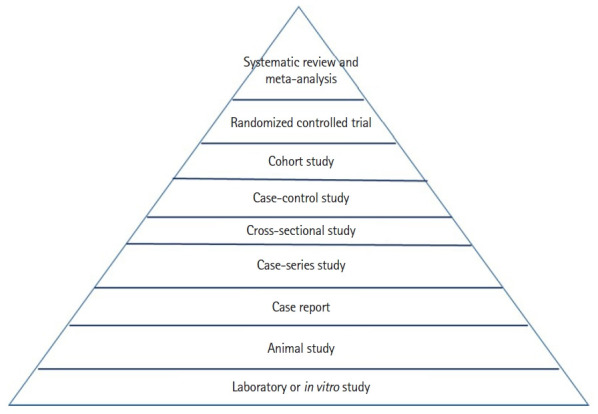
Levels of evidence pyramid. Adapted from Murad et al. [16], available under the Creative Commons license.

The most important point in the definition of EBM is “current best evidence.” In general, several types of study designs are used to investigate the causal relationship between a risk factor and a certain disease in epidemiology or to evaluate the efficacy and safety of an intervention, such as a pharmaceutical drug or a certain treatment method in medicine. There is also a hierarchy in terms of levels of evidence among different study designs. In 1979, a report by the Canadian Task Force on the Periodic Health Examination [15] first proposed levels of evidence regarding recommendations for the examination. For example, the task force graded the effectiveness of interventions based on the quality of the evidence as follows: grade 1, evidence is obtained from at least one properly randomized controlled trial (RCT); grade 2-1, evidence is obtained from well-designed cohort or case-control studies; grade 2-2, evidence is obtained from comparisons between times or places with or without the intervention or dramatic results in uncontrolled experiments; and grade 3, evidence derives from the opinions of respected authorities, based on clinical experience and so forth [15]. Since then, various versions of the “levels of evidence pyramid” have been described, and a standard levels of evidence pyramid has been established [16] (Fig. 1): weaker study designs such as laboratory studies and animal studies are located on the bottom of the pyramid, and then case reports, case-series studies, cross-sectional studies, case-control studies, cohort studies, and RCTs are located in the middle of the pyramid (middle levels of evidence). Thus, in general, when there are inconsistent findings on a certain research topic across various study designs, it can be concluded that study designs located in the higher rows of the pyramid are more reliable, valid, and preferred in practice than those in the lower rows of the pyramid. Because systematic reviews and meta-analyses are located at the top of the pyramid, they generally provide the highest level of evidence among all types of study designs.

## Procedure of a meta-analysis

This review briefly summarizes how to conduct a meta-analysis, as follows.

### Define a research topic

A well-defined research topic is the most important starting point for an excellent systematic review with meta-analysis. Even if a certain research topic seems fantastic or excellent, conducting a meta-analysis is impossible or not meaningful if there are no published individual studies or very few studies (usually fewer than 5 or so). Theoretically, if at least 2 individual studies are published on a certain topic, a meta-analysis is possible. However, a meta-analysis with only 2 studies would generally have too small a sample size to draw a conclusion and provide any new knowledge. Thus, in general, if the number of individual studies published on the same topic is at least 5 or, if possible, more than 10, it would be appropriate to conduct a meta-analysis. It is also preferable if no meta-analysis has yet been published on a certain topic. However, in most cases, at least one meta-analysis on a certain topic already exists. This should not deter researchers from conducting further meta-analyses on the topic, especially if subsequent individual studies on the same topic have been published since the most recent meta-analysis, the conclusions of the planned meta-analysis are expected to be different from those of the previous ones, or in some cases, it is considered meaningful to replicate and confirm previous findings.

### Select a study design

In the field of medicine, the most common types of study designs used for meta-analysis are case-control studies, cohort studies, and RCTs. Thus, it is very important to identify which type(s) of study design will be selected for a given research question at the very beginning of research.

### Search the literature in electronic databases

The core databases used for meta-analysis in the field of medicine are PubMed (Medline), Embase (Excerpta Medica database), and CENTRAL (Cochrane Central Register of Controlled Trials). Among them, CENTRAL is a database for bibliographic reports of RCTs taken from published and unpublished sources such as CINAHL (Cumulative Index to Nursing and Allied Health Literature), ClinicalTrials.gov, and the International Clinical Trials Registry Platform from the World Health Organization (WHO), as well as PubMed and Embase. Thus, if researchers plan to conduct a meta-analysis of observational studies, such as case-control studies or cohort studies, they do not need to search CENTRAL. Instead, searching the 2 core databases of PubMed and Embase suffices.

When searching a database, appropriate search keywords (terms) related to the research topic should be selected and combined. Both the National Library of Medicine (NLM) Medical Subject Headings (MeSH) terms and a wide range of free-text search terms are used in order to identify as many relevant articles as possible. Usually, 2 types of search terms, such as a keyword for an intervention or an exposure variable and a keyword for an outcome variable, are combined. For example, if the topic is the association between the intake of vitamin C and the risk of lung cancer, by using Boolean operators for all possible MeSH and free-text terms, the following search terms can be used: (vitamin C OR ascorbic acid) AND (lung cancer OR lung neoplasm).

### Select relevant studies

The PICO (patient, problem, or population; intervention; comparison, control or comparator; and outcome) criteria regarding the research topic are used for selecting relevant studies. The types of study designs, such as case-control studies, cohort studies, or RCTs, should be determined. In general, the selection process is conducted stepwise, with an initial screening of titles and abstracts followed by a final full-text screening. Based on the predetermined selection criteria for the individual studies that will be included in the meta-analysis, at least 2 of the authors should independently assess the eligibility of the studies and select relevant studies.

### Conduct a meta-analysis

In meta-analyses in the field of medicine, the most commonly used effect sizes are odds ratios (ORs) or relative risk (RRs) for dichotomous variables and weighted mean differences (WMDs) or standardized mean differences (SMDs) for continuous variables. In the case of dichotomous variables, an arrangement of 4 cells in a 2×2 table in each RCT is used to combine the results of the included studies in meta-analyses of RCTs, whereas an adjusted OR in each case-control study or an RR in each cohort study with lower and upper limits of the 95% CI are used in meta-analyses of observational studies. For continuous variables, the WMD is used for outcomes on the same scales, such as blood pressure (mmHg) or serum glucose levels (mg/dL) across studies, and the SMD is used for the outcomes on different scales, such as fatigue score measures using different questionnaire-based tools across studies. A meta-analysis involves combining these individual effect sizes to estimate the overall or summary effect size. In general, the common software programs used for performing meta-analysis to estimate the overall effect size are Stata (Stata (Corp.), RevMan5 (Cochrane), R (R Foundation for Statistical Computing), Excel (Microsoft (Corp.), Comprehensive Meta-analysis (Biostat (nc.), and IBM SPSS (IBM (Corp.). In addition to the main analysis on a specific research topic, subgroup meta-analyses by various factors such as sex/gender, dosage of a certain drug, follow-up period, study quality, study region, funding source, and other variables can be performed as appropriate for a given topic.

## Assessment of findings from a meta-analysis

### Interpretation of a forest plot

The main findings of meta-analyses are presented by creating a forest plot, also known as a blobbogram, which is a graphical display of individual results from studies included in the analysis and an overall combined result.

**Fig. 2.**
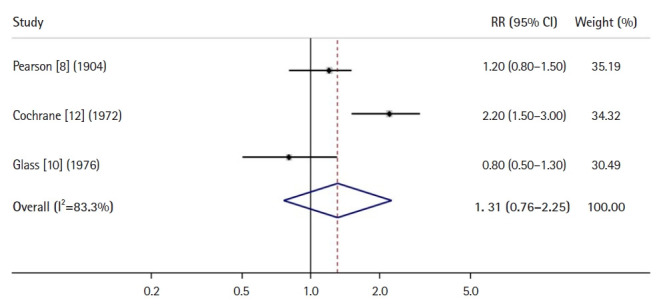
An example forest plot using a relative risk (RR) from a meta-analysis of 3 cohort studies or randomized controlled trial. CI, confidence interval.

Fig. 2 shows an example of a forest plot using RRs for a dichotomous variable from a meta-analysis of 3 cohort studies or RCTs [8, 10, 12]. The left column lists the names of individual studies included in the analysis using the year and family name of the first author of each study in chronological order, and the center column is a plot of individual results, with a square representing each RR and a horizontal line representing its CI. The overall combined result is shown using a diamond, the right and left vertices of which represent its CI. A central vertical line indicates no effect or association. Thus, because the CI (0.76 to 2.25) for the overall RR, as shown in Fig. 2, overlaps with the vertical line indicating the number 1, it is interpreted that there is no significant association between 2 variables (e.g., a risk factor or an intervention vs. an outcome of a disease). The left column lists the values for RRs with their CIs in individual studies, an overall RR with its CI in combined studies, and weights as percentages, which are usually proportional to the sample size of each study.

**Fig. 3.**
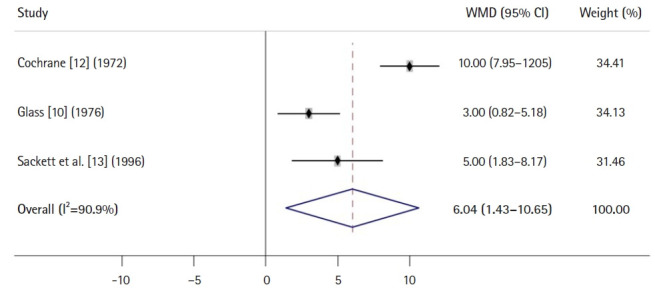
An example forest plot using a weighted mean difference (WMD) from a meta-analysis of 3 randomized controlled trials. CI, confidence interval.

Fig. 3 shows an example of a forest plot using a WMD for a continuous variable from a meta-analysis of 3 RCTs [10,12,13]. Because the CI (1.43 to 10.65) for the overall WMD does not overlap with the vertical line indicating the number 0, it is interpreted that a certain intervention or treatment group has a significant effect of 6.04 (actual value, e.g., mg/dL for blood glucose levels) compared with a control group.

**Fig. 4.**
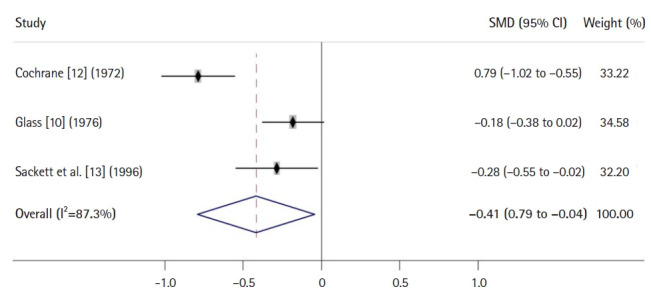
An example forest plot using a standardized mean difference (SMD) from a meta-analysis of 3 randomized controlled trials. CI, confidence interval.

Fig. 4 shows an example of a forest plot using an SMD for a continuous variable from a meta-analysis of 3 RCTs [10,12,13]. Because the CI (–0.79 to –0.04) for the overall SMD does not overlap with the vertical line indicating the number 0, it is interpreted that a certain intervention or treatment group has a significant effect compared with a control group. Here, because the SMD used for the outcome on the different scales across studies is an effect size standardized to a uniform scale by dividing a mean difference between 2 groups by the pooled standard deviation from 2 groups, the value –0.41 is not an actual one, but a measure of distance or difference between 2 groups. In general, according to Cohen’s suggestion, an SMD of 0.2 is interpreted as indicating a small effect or difference, an SMD of 0.5 is interpreted as a medium effect, and an SMD of 0.8 or higher is considered indicative of a large effect.

### Interpretation of a funnel plot

A funnel plot, which is mainly used to examine the existence of publication bias, is also important for interpreting a meta-analysis. A funnel plot is a scatter plot of the effect size, such as OR or RR, on the x-axis against a measure of the study precision, such as each study’s sample size or standard error, on the y-axis. Studies with higher precision (e.g., larger studies) have a small standard error, located towards the top, and are placed near the average, whereas smaller studies are scattered widely at the bottom. Thus, the plot resembles a symmetrical inverted funnel. However, visually apparent asymmetry or a P-value of <0.05 from the Egger indicates the existence of publication bias. Other than publication bias, possible sources of funnel plot asymmetries are other reporting biases (e.g., selective outcome reporting), poor methodological quality, true heterogeneity, and chance [17].

### Interpretation of heterogeneity

Any kind of variability across studies included in the meta-analysis is called heterogeneity. There are 3 types of heterogeneity: clinical, methodological, and statistical. Statistical heterogeneity, which is variability or difference in effect sizes across studies and might be a consequence of clinical or methodological heterogeneity, or both, is assessed by using the Cochran Q statistic or I^2^ index. Usually, a P-value of less than 0.1 for the Q statistic is used to provide evidence of heterogeneity. As a rough guide to interpretation of the I2 index, percentages of 25%, 50%, and 75% indicate low, medium, and high heterogeneity, respectively [18]. I^2^ values greater than 50% are considered as showing substantial heterogeneity [19].

## Important tips for reviewing systematic reviews and meta-analyses

When reviewing systematic reviews and meta-analyses, reviewers should consider the following important tips, which were originally presented on the BMJ website and have been adapted for the review of systematic review and meta-analysis articles [20].

### Originality

Originality is one of the most important criteria for good research. Thus, reviewers should assess whether the submitted research article presenting a systematic review and meta-analysis adds new knowledge to what is already known and also whether the systematic review and meta-analysis design is appropriate and adequate to answer the research question. They should describe the originality of the work and cite relevant references to support their comments on its originality. In order to check originality, they should search the previous literature to identify systematic reviews and meta-analyses on the same topic published in the electronic core databases. As mentioned earlier, even if multiple meta-analysis articles on the same topic have already been published, if subsequent individual studies on the same topic have been published since the most recent meta-analysis, or if the findings or conclusions of the current meta-analysis would be different from those of the previous ones, the work might be of value.

### Importance of the work

Reviewers also should assess the importance of the work. For example, if a systematic review and meta-analysis article is submitted to a general medical journal, they should assess whether it matters to the readers of the journal such as medical doctors, clinicians, medical researchers, or professors in medical colleges. That is, reviewers should evaluate whether the journal is the right place for the work.

### Database search

As described earlier, it is recommended to search 3 core electronic databases—PubMed (Medline), Embase, and CENTRAL—for the purpose of meta-analysis of RCTs in the field of medicine. Searching just one electronic database, such as PubMed, is insufficient. For a meta-analysis of observational studies, such as case-control studies or cohort studies, it suffices to search PubMed and Embase.

### Selection of studies (inclusion and exclusion criteria)

It is important to evaluate which studies are selected and included in the meta-analysis. As mentioned earlier, it is important for authors to use the PICO framework to select relevant studies on the research topic. Above all, reviews should evaluate whether the type of study design (e.g., case-control studies, cohort studies, or RCTs) as an inclusion criterion is appropriate and adequate to answer the research question. Reviewers should also determine whether the intervention, comparison, and outcome measures are appropriate. The description of exclusion criteria should also be checked. In general, common exclusion criteria are nonhuman studies, studies with duplicate or overlapping data, nonoriginal studies, and non-English publications.

### Subgroup analyses by various factors

It is important for the authors of a systematic review and meta-analysis to perform subgroup meta-analyses according to various factors. In many cases, even if a main meta-analysis including all the studies does not find any significant association or effect, subgroup meta-analyses according to some important or interesting factors might show significant findings. For example, in subgroup meta-analyses by study quality, those with high quality might show no significant effect of a certain intervention, while those with low quality show significant effects. Additionally, a funding source from a pharmaceutical company constituting a conflict of interest might affect the results of an RCT for the effect of a new drug. Other important factors considered in subgroup meta-analyses are the type of participants (age, sex/gender, race/ethnicity), type of intervention or risk factor, type of comparison, type of outcome, dosage, and the intervention or follow-up period.

### Interpretation based on levels of evidence

It would be ideal to conduct a meta-analysis of individual studies with the same study design, such as a meta-analysis of case-control studies, a meta-analysis of cohort studies, or a meta-analysis of RCTs. However, it is possible to combine 2 different study designs, such as case-control studies and cohort studies, in one meta-analysis, if the study designs are similar (e.g., observational studies). In such cases, caution is required in the interpretation of the results of the meta-analysis. Suppose that a meta-analysis of combined case-control studies and cohort studies shows a significant association between a risk factor and a disease, but the subgroup meta-analysis of cohort studies finds no significant association between them, while that of case-control studies does show a significant association. Based on the levels of evidence, the correct interpretation of these findings should be that there is no significant association between the variables because cohort studies generally provide a higher level of evidence than case-control studies. For example, a large meta-analysis of 222 articles in 2013 including both case-control and cohort studies concluded that light alcohol drinking (up to one drink per day) increases the risk of oral cavity and pharynx, esophagus, and female breast cancer [21]. This meta-analysis contributed to the revision of the European Code Against Cancer 4th edition, published in 2015, which newly recommended that “Not drinking alcohol is better for cancer prevention.” However, in my opinion, the authors of the meta-analysis made an erroneous conclusion because there was no significant association between light alcohol drinking and the risk of oral cavity and pharynx cancer or esophagus cancer in the subgroup meta-analysis of cohort studies, although a significantly increased risk was found for those cancers in the meta-analysis of only case-control studies [22]. When there is a difference in findings between case-control studies and cohort studies, the findings from cohort studies are generally more reliable than those from case-control studies.

### Other topics, including common mistakes

A common mistake made by authors of meta-analyses is to combine the same dataset that is duplicated in multiple publications from a study. Another is to combine non-independent data partly shared or overlapping among study participants. It is possible to combine each effect size, such as an OR or an RR in each sex/gender or different datasets of the completely independent subgroups in a study. Reviewers also should evaluate the following: Does the introduction section well describe the backgrounds and aims of the systematic review and meta-analysis? Are the findings of the previous literature on the same research topic, such as individual studies and systematic review and meta-analysis articles, well summarized? Are the overall methods adequately described? Does the Results section answer the research question reliably? Are the findings from the analysis well presented in Tables and Figures? Does the Discussion section clearly address the main findings, comparisons with the previous literature, possible mechanisms underlying the association between the risk factors or interventions and outcomes, and limitations of the work? Is the conclusion clear? Are the references up to date and relevant?

## Conclusion

I briefly summarized what systematic reviews and meta-analyses are and how to review and assess systematic review and meta-analysis articles in the field of medicine. I hope this review provides useful assistance regarding how to read, interpret, and evaluate these articles.

## Figures and Tables

**Figure f5-jeehp-20-24:**
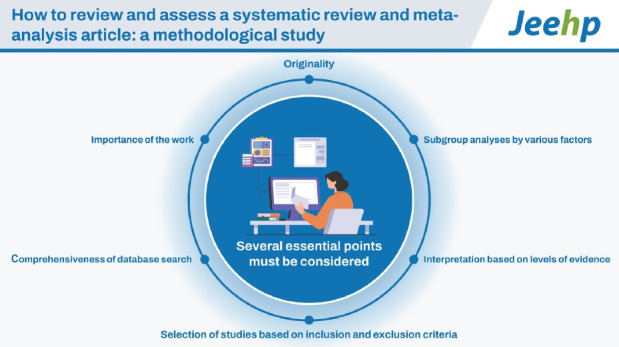


## Supplementary materials

Supplement 1. Audio recording of the abstract.
